# *Bifidobacterium breve* Exopolysaccharide Blocks Dendritic Cell Maturation and Activation of CD4^+^ T Cells

**DOI:** 10.3389/fmicb.2021.653587

**Published:** 2021-06-16

**Authors:** Ana Hickey, Panagiota Stamou, Sreeram Udayan, Ana Ramón-Vázquez, Maria Esteban-Torres, Francesca Bottacini, Jerzy Adam Woznicki, Owen Hughes, Silvia Melgar, Marco Ventura, Douwe Van Sinderen, Valerio Rossini, Ken Nally

**Affiliations:** ^1^APC Microbiome Ireland, University College Cork, Cork, Ireland; ^2^School of Biochemistry and Cell Biology, University College Cork, Cork, Ireland; ^3^School of Microbiology, University College Cork, Cork, Ireland; ^4^Luminex Corporation, Austin, TX, United States; ^5^Life Sciences and Environmental Sustainability, University of Parma, Parma, Italy

**Keywords:** *Bifidobacterium breve*, exopolysaccharides, host immune responses, macrophages, dendritic cells, antigen presentation

## Abstract

Exopolysaccharide (EPS) is a bacterial extracellular carbohydrate moiety which has been associated with immunomodulatory activity and host protective effects of several gut commensal bacteria. *Bifidobacterium breve* are early colonizers of the human gastrointestinal tract (GIT) but the role of EPS in mediating their effects on the host has not been investigated for many strains. Here, we characterized EPS production by a panel of human *B. breve* isolates and investigated the effect of EPS status on host immune responses using human and murine cell culture-based assay systems. We report that *B. breve* EPS production is heterogenous across strains and that immune responses in human THP-1 monocytes are strain-specific, but not EPS status-specific. Using wild type and isogenic EPS deficient mutants of *B. breve* strains UCC2003 and JCM7017 we show that EPS had strain-specific divergent effects on cytokine responses from murine bone marrow derived macrophages (BMDMs) and dendritic cells (BMDCs). The *B. breve* UCC2003 EPS negative (EPS^–^) strain increased expression of cytokine genes (*Tnfa, Il6, Il12a*, and *Il23a*) relative to untreated BMDCs and BMDCs treated with wild type strain. *B. breve* UCC2003 and JCM7017 EPS^–^ strains increased expression of dendritic cell (DC) activation and maturation marker genes (*Cd80, Cd83*, and *Cd86*) relative to untreated BMDCs. Consistent with this, BMDCs co-cultured with *B. breve* UCC2003 and JCM7017 EPS^–^ strains engineered to express OVA antigen activated OVA-specific OT-II CD4^+^ T-cells in a co-culture antigen-presentation assay while EPS proficient strains did not. Collectively, these data indicate that *B. breve* EPS proficient strains use EPS to prevent maturation of DCs and activation of antigen specific CD4^+^ T cells responses to *B. breve*. This study identifies a new immunomodulatory role for *B. breve* EPS and suggests it may be important for immune evasion of adaptive immunity by *B. breve* and contribute to host-microbe mutualism.

## Introduction

Bifidobacteria elicit protective immunomodulatory effects on murine and human hosts including reduction of gastrointestinal (GI) diseases and potentially systemic effects (Sivan et al., 2015; Sarkar and Mandal, 2016; Riedel, 2018). However, mechanisms underpinning these effects are still poorly understood (Fischbach, 2018; Round and Palm, 2018). Identification and characterization of specific bifidobacterial microbe-associated molecular patterns (MAMPs), their corresponding host pattern recognition receptors (PRRs) (Round et al., 2011) and associated immune cell responses is needed in order to understand proposed immunomodulatory properties of bifidobacteria. One of these candidate bifidobacterial MAMPs is EPS, a surface-associated glycan which is used by pathogens and other microbiota members to manipulate host responses (Castro-Bravo et al., 2018b). There is large structural diversity in EPS polymers produced by bifidobacteria, even between strains of the same species (Bottacini et al., 2014; Hidalgo-Cantabrana et al., 2014; Ferrario et al., 2016). The genes responsible for EPS biosynthesis are clustered within one or two transcriptional units. Variations in monosaccharide components and quantities of such, in addition to differences in glycosidic linkages and the degree of branching have been observed for different bifidobacteria strains (Leivers et al., 2011; Hidalgo-Cantabrana et al., 2014; Inturri et al., 2017; Castro-Bravo et al., 2018a). This variation could, in principle, lead to a vast number of distinct EPS structures and, theoretically to different immunomodulatory effects on the host. Reports indicate that purified EPS from two different bifidobacterial species can be detected by host innate immune PRRs – toll like receptor (TLR) 2 and TLR4 (Schiavi et al., 2018; Castro-Bravo et al., 2019). Bifidobacterial EPS has also been shown to ameliorate inflammation in murine pulmonary and intestinal models of inflammation (Hidalgo-Cantabrana et al., 2016; Schiavi et al., 2018). Previously, our group showed that EPS deficient *B. breve* UCC2003 had profound immunomodulatory effects on the host with an increase in pro-inflammatory cytokines interferon (IFN)-γ, tumor necrosis factor (TNF)-α and IL-12 detected from splenocytes cultured with the bacteria. These effects were associated with an increase in neutrophils, macrophages, natural killer cells, B cells and IFN-γ^+^, TNF-α^+^, and IL-12^+^ T cells (Fanning et al., 2012). Furthermore, the administration of EPS deficient *B. breve* to mice increased susceptibility to *Citrobacter rodentium* infection and reduced persistence of *B. breve* in the murine GIT (Fanning et al., 2012). Although many studies have used purified EPS to characterize host responses, this approach is susceptible to contamination with additional MAMPs derived from components of bacterial cell walls, membranes, or even cytoplasms (Alhudhud et al., 2018). An alternative reductionist approach is to investigate the effects of EPS using EPS deficient isogenic mutants of EPS producers and compare immune cell responses to both WT EPS proficient and mutant EPS deficient strains. EPS deficient mutants have been created for several bifidobacterial strains such as *Bifidobacterium longum* ssp. *longum* 35624 (Schiavi et al., 2016), *B. longum* 105-A (Tahoun et al., 2017), *Bifidobacterium breve* UCC2003 (Fanning et al., 2012), and *B. breve* JCM7017 (Alhudhud et al., 2018). Another consideration when investigating EPS-mediated effects is to use experimental cell-based systems from different host species as bifidobacteria are found and isolated from various ecological niches (Turroni et al., 2018). In this study, we analyzed a panel of 12 *B. breve* strains, which were isolated from humans, to assess EPS production. Two of these strains had matching isogenic EPS deficient mutant strains as previously described (*B. breve* UCC2003 and JCM7017) (Fanning et al., 2012; Alhudhud et al., 2018) and were used to determine the contribution of EPS to host immune cell responses. We found that *B. breve* EPS production was required to prevent maturation of dendritic cells and dendritic cell-mediated activation of CD4^+^ T cells responses.

## Materials and Methods

### Bacterial Culture Conditions and Plasmid Transformation

All bacterial strains used in this study are listed in Table 1. Strains were routinely cultured in reinforced clostridial medium (RCM; Oxoid cat. CM0149), sub-cultured into de Man Rogosa and Sharpe Medium (MRS; Becton Dickonson Difco cat. 288130) supplemented with 0.05% cysteine-HCl (Sigma) and incubated anaerobically at 37°C. Culture of bifidobacterial strains for growth curves was carried out in modified MRS (mMRS) medium, which was prepared from first principles (De Man et al., 1960) and supplemented with 0.05% cysteine-HCl (Sigma) and 1% glucose (Sigma). Cultures containing either *B. breve* UCC2003 EPS^–^ or *B. breve* JCM7017 EPS^–^ mutants were supplemented with 0.01 mg/mL tetracycline (Sigma) and cultures containing bacteria transformed with the pMG-mCherry-OVA plasmid were supplemented with 0.01 mg/mL chloramphenicol (Sigma). The pMG-mCherry-OVA plasmid was constructed from the pMG-mCherry plasmid as described by Grimm et al. (2014). Transformation of the pMG-mCherry-OVA plasmid into *B. breve* strains was achieved by electroporation with subsequent screening for chloramphenicol-resistant colonies on RCM agar plates containing 0.01 mg/mL chloramphenicol (Sigma). Further confirmation of mCherry-OVA expression was carried out by flow cytometry for mCherry expression (excitation at 545 ± 30 nm, emission at 610 ± 75 nm) using the BD FACSAria III Fusion Cell sorter, polymerase chain reaction and western blot. Flow cytometry data was further analyzed using FCS Express version 5 software (*De Novo*).

**TABLE 1:** List of B. breve strains used in study, their origin of isolation and genome accession number.

**Strain**	**Origin**	**Accession number**	**References**
UCC2003	Human infant feces	NC_020517	Bottacini et al., 2014
UCC2003_EPSneg	Mutant made in UCC		Fanning et al., 2012
JCM7017	Human adult feces	CP006712	Bottacini et al., 2014
JCM7017_EPSneg	Mutant made in UCC		Alhudhud et al., 2018
JCM7019	Human infant feces	CP006713	Bottacini et al., 2014
215W447A	Human infant feces	CP021558	Bottacini et al., 2017
017W439A	Human infant feces	CP021554	Bottacini et al., 2017
082W48	Human infant feces	CP021555	Bottacini et al., 2017
689b	Human infant feces	CP006715	Bottacini et al., 2014
NCTC 11815	Human infant feces	BBAO00000000	Bottacini et al., 2014
139W4	Human infant feces	CP021556	Bottacini et al., 2017
NCFB 2258	Human infant feces	CP006714	Bottacini et al., 2014
180W83	Human infant feces	CP021557	Bottacini et al., 2017
B12L	Human milk	CP006711	Bottacini et al., 2014

### Electron Microscopy

Bacteria were prepared as described. Materials were directly adsorbed onto a carbon film membrane on a 300-mesh copper grid, stained with 1% uranyl acetate, dissolved in distilled water, and dried at room temperature. Grids were examined with Hitachi HT7700 electron microscope operated at 80 kV (Elexience – France), and images were acquired with a charge-coupled device camera (AMT). This work was carried out at the facilities and expertise of MIMA2 MET – GABI, INRA, Agroparistech (78352 Jouy-en-Josas, France).

### *In silico* and *in vitro* Analysis of EPS Production

*In silico* analysis of *B. breve* fully sequenced strains was carried out using comparative genome analysis according to a previously described method (Bottacini et al., 2018). Whole genome sequences of 12 *B. breve* strains were retrieved from the National Centre for Biotechnology Information (NCBI) database. The genomic region previously described as associated to EPS biosynthesis in *B. breve* UCC2003 (Fanning et al., 2012; Bottacini et al., 2017), encoded between a priming glycosyl transferase (Bbr_0430) and a chain length determinant (Bbr_0474) was used as starting point for predicting homologous EPS-encoding regions in the 12 *B. breve* strains here analyzed. Presence/absence of homologous genes was retrieved by comparative genome analysis using “all-against-all,” bi-directional BLASTP alignments49 (cut-off: *E*-value < 0.0001, with at least 50% identity across at least 50% of either protein sequence). Based on previous descriptions for the *B. breve* spp., each strain was classified as an EPS “producer” or “non-producer” based on the presence of: (a) an EPS locus larger than 30 Kb, (b) at least three glycosyltransferase (GTF)-encoding genes within the predicted EPS locus, (c) a priming glycosyltransferase, (d) a gene encoding a flippase, (e) a chain length determinant. EPS status was assessed *in vitro* by a sedimentation assay as previously described (Fanning et al., 2012; Tahoun et al., 2017). Briefly, optical density measurements (OD_600 nm_) of *B. breve* strains and EPS^–^ mutants were taken over a 6 h time period grown in mMRS medium, which was prepared from first principles (De Man et al., 1960) and supplemented with 0.05% cysteine-HCl and 1% glucose, lactose or maltose (Sigma) without agitation. A drop in OD values due to sedimentation is a characteristic of EPS non-producers.

### Mice

Mice used for this study were 8–12 weeks old from a C57BL/6 background. WT C57BL/6 mice for isolation of bone marrow and generation of BMDMs and BMDCs were purchased from Envigo (Oxfordshire, United Kingdom). OT-II mice were acquired from Jackson Laboratories (Bar Harbor, United States) and breeding was maintained in house. All mice were housed in UCC Biological Services Unit under specific pathogen free (SPF) conditions. Standard housing conditions were maintained with 12 h darkness and 12 h light, temperature controlled at 21°C and 50% humidity. Animals were fed standard chow pellets and water was given *ad libitum*. The animal work was performed in accordance with EU legislation, in accordance with EU Directive 2010/63/EU, for the protection of animals used for scientific purposes and approved by the Animal Experimentation Ethics Committee of University College Cork, Euthanasia Only Application ID 2018/007.

### THP-1 Human Monocytic NF-κB Reporter Cells

THP1-XBlue^TM^ and THP1-XBlue^TM^-defMyD (MyD88 deficient) NF-κB reporter cell lines were purchased from Invivogen. Cells were routinely cultured using Roswell Park Memorial Institute medium (RPMI 1640; Sigma cat. R8758) supplemented with 10% Fetal Bovine Serum (FBS; Sigma), 1% Penicillin-Streptomycin (P/S; Sigma), 100 μg/mL Normocin (Invivogen) and 200 μg/mL Zeocin (Invivogen). For the THP1-XBlue^TM^-defMyD cells 100 μg/mL Hygromycin B Gold (Invivogen) was added to the medium. Cells were continuously maintained at a density between 7 × 10^5^ cells/mL and 2 × 10^6^ cells/mL. NF-κB activity in THP-1 reporter cell lines was assayed using Quanti-blue (Invivogen cat. rep-qb1), as per manufacturer’s instructions.

### GM-CSF-Derived Bone Marrow Derived Macrophages and Dendritic Cells

Bone marrow isolation was carried out using aseptic techniques. This method was based on the work of Helft et al. (2015). Briefly, bone marrow was isolated from the femurs and tibias of selected mice by flushing using RPMI and filtered through a 70 μm strainer. Cells were collected by centrifugation at 200 × *g* at 4°C for 10 min. The cell pellet was resuspended in differentiation media – RPMI containing 10% FBS, 1% P/S and 20 ng/mL GM-CSF (PeproTech cat. 315-03). Cells were counted and 10^7^ cells were seeded per well in 6 well plates with each well containing 4 mL of differentiation media. On day 2 half of the content was collected from each well, centrifuged and cells were resuspended in RPMI containing 10% FBS, 1% P/S and 40 ng/mL GM-CSF and added back to the 6 well plates. On day 3, all content from each well was harvested, centrifuged and cells were resuspended in RPMI containing 10% FBS, 1% P/S and 20 ng/mL GM-CSF before being added back to the 6 well plates. Both suspension cells (containing the DC fraction) and adherent cells (containing the macrophage fraction) were collected on day 6 and prepared for sorting. Prior to collection, adherent cells were harvested using Stempro Accutase (Thermo Scientific). Cells were stained using anti-mouse CD11c Alexa Fluor 700 (BioLegend cat. 117320), anti-mouse I-A/I-E fluorescein isothiocyanate (BioLegend cat. 107606), anti-mouse CD11b R-phycoerythrin cyanine 7 (BioLegend cat. 101216) and anti-mouse CD115 Allophycocyanin (BioLegend cat. 135510). Cells were then analyzed using the BD FACSAriaIII Fusion Cell sorter and sorted into pure populations of BMDMs and BMDCs based on a gating strategy modified from Helft et al. (2015). Data was further analyzed using FCS Express version 5 software (*De Novo*).

### Co-culture of Mammalian Cells and Bifidobacteria

For all experiments, after an overnight culture of bacteria an OD_6__00 n__m_ of 1 corresponded to 10^9^ CFU of bacteria. Bacteria were washed twice in sterile PBS and resuspended in the appropriate mammalian cell culture medium with no antibiotics and concentration was adjusted as required. For cytokine and NF-κB activity experiments, BMDMs, BMDCs and THP-1 monocytes were seeded in 96 well plates at a density of 5 × 10^4^ cells/well in RPMI with 10% FBS and incubated with bacteria at a multiplicity of infection of 10:1 bacteria:cells. For BMDM and BMDCs experiments 100 ng/mL of LPS-B5 (Invivogen cat. tlrl-b5lps) and 5 μM of ODN 1585 CpG (Invivogen cat. tlrl-1585) were added as a positive controls, respectively. Co-cultures were incubated for 24 h at 37°C in 5% CO_2_. After 24 h, cells were collected by centrifugation and supernatants analyzed for NF-κB activity (THP-1 cells only). For reverse transcription-quantitative polymerase chain reaction (RT-qPCR) BMDMs or BMDCs were seeded at 2.5 × 10^5^ cells were seeded per well in 12 well plates in RPMI with 10% FBS and incubated with bacteria at a multiplicity of infection of 10:1. 100 ng/mL of LPS-B5 (Invivogen cat. tlrl-b5lps) was used as a positive control in BMDM cultures and 5 μM of ODN 1585 CpG (Invivogen cat. tlrl-1585) was used as a positive control in BMDCs cultures. Co-cultures were incubated for 4 or 8 h at 37°C in 5% CO_2_. After 4 or 8 h, BMDMs were washed with ice cold PBS once and lysed using RLT lysis buffer (Qiagen). BMDCs were transferred to 1.5 mL tubes and centrifuged for 7 min at 400 × *g* at 4°C. Supernatants were removed and cells lysed in RLT lysis buffer. Both BMDM and BMDs lysates were immediately stored at −80°C until total RNA was extracted.

### Cytokine ELISAs

BMDM and BMDCs co-culture supernatants were analyzed for secreted cytokines TNF-α and IL-10 using mouse TNF-alpha DuoSet ELISA (R&D Systems cat. DY410) and mouse IL-10 DuoSet ELISA (R&D Systems cat. DY417) as per manufacturer’s instructions. Supernatants from T cell and BMDM or BMDCs co-cultures were analyzed for secreted cytokines TNF-α and IL-2 using mouse TNF-alpha DuoSet ELISA (R&D Systems cat. DY410) and mouse IL-2 DuoSet ELISA (R&D Systems cat. DY402) as per manufacturer’s instructions. TNF-α production by THP-1 reporter cell lines was measured in supernatants from these experiments using human TNF-α ELISA Duoset (R&D Systems cat. DY210).

### RT-qPCR

RNA was isolated from BMDMs and BMDCs using RNeasy Micro Kit (Qiagen cat. 74004) which included DNase treatment. Total RNA was quantified using Qubit RNA high sensitivity kit (Biosciences cat. Q32852). Complementary DNA (cDNA) was synthesized from 100 ng of total RNA using Transcriptor Reverse Transcriptase (Roche) and random hexamer primers (Roche) as per manufacturer’s instructions. qPCR assays were designed using the Roche Universal Probe Library Assay Design Centre (Table 2). qPCR assays were carried out by using SensiFAST Probe No-ROX Kit (Bioline), 500 nM primers, 250 nM corresponding ProbeLibrary probe from Universal ProbeLibrary (Roche) and 2 ng of cDNA. PCR reactions were run on a LightCycler 480 instrument (Roche) and the 2^–ΔΔCT^ method (Mar et al., 2009; McCall et al., 2014) was used to calculate relative gene changes in gene expression compared to β-actin which served as a housekeeping gene (Livak and Schmittgen, 2001).

**TABLE 2:** List of genes, Universal ProbeLibrary (UPL) probes and primers used for RT-qPCR.

**Gene**	**Probe**	**Forward primer**	**Reverse primer**
Il10	41	cagagccacatgctcctaga	tgtccagctggtcctttgtt
Tnfa	49	tcttctcattcctgcttgtgg	ggtctgggccatagaactga
Il6	6	gctaccaaactggatataatcagga	ccaggtagctatggtactccagaa
Il12a	62	ccaggtgtcttagccagtcc	gcagtgcaggaataatgtttca
Il12b	27	gactccaggggacaggcta	ccaggagatggttagcttctga
Il23a	19	tccctactaggactcagccaac	agaactcaggctgggcatc
H2/MHCII	110	ctctgattctgggggtcct	accataggtgcctacgtggt
Cd80	91	ttcgtctttcacaagtgtcttca	tgccagtagattcggtcttca
Cd86	107	gaagccgaatcagcctagc	cagcgttactatcccgctct
Adhl1a2	33	catggtatcctccgcaatg	gcgcatttaaggcattgtaac
Cd274	101	gttgttcctcattgtagtgtcca	cacatttctccacatctagcattc
Pdcd1lg2	17	gcatgttctggaatgctcac	ctttgggttccatccgact
Cd83	29	accgtggttctgaaggtgac	caaccagagagaagagcaacac
β-actin	64	ctaaggccaaccgtgaaaag	accagaggcatacagggaca

### Western Blotting

After an overnight culture, bacterial cells were resuspended in ice-cold 10 mM Tris-HCl pH 8, transferred to tubes containing glass microbeads and bead-beated three times. Lysates were centrifuged at maximum speed for 15 min at 4°C and supernatants were transferred to fresh 1.5 mL tubes. Protein was quantified using Pierce BCA Protein Assay Kit (Thermo Scientific) as per manufacturer’s instructions. Fifty μg of protein for each sample was standardized and the appropriate volume of 4× LDS sample buffer and 10× sample reducing agent (Novex) was added to each sample. Protein was denatured by heating at 70°C for 10 min. Samples were separated on a 4–12% Bis-Tris Plus Gel (Invitrogen) alongside SeeBlue Plus2 Prestained Standard ladder (Invitrogen) and transferred onto a PVDF membrane (Millipore) for antibody detection. Membranes were stained with Ponceau S to confirm protein transfer, blocked for 1 h rocking at room temperature with 5% milk powder (Sigma) dissolved in 0.05% Tween-20 TBS (TBST), washed three times with TBST and incubated rocking overnight at 4°C with anti-OVA (Abbiotec cat. 250803) in 5% milk in TBST. Membranes were washed with TBST and incubated with HRP-conjugated goat anti-rabbit secondary antibody (Sigma) in 5% milk in TBST. Membranes were washed with TBST and WesternBright substrate (Advansta) was added. Images were acquired using Fujifilm LAS-3000 instrument and software.

### Co-culture Antigen Presentation Assay With Bacterial – BMDC– CD4^+^ T Cells

5 × 10^4^ sorted BMDCs were seeded per well in 96 well plates in RPMI with 10% FBS but no antibiotics. Bacteria expressing mCherry-OVA were added at ratio 10:1. OVA protein (Sigma) was added at a final concentration of 150 μg/mL as a positive control. The cells were incubated with OVA protein or bacteria for 8 h at 37°C in a cell culture incubator at 5% CO_2_. CD4^+^ T cells were isolated from OT-II mice by spleen homogenization. Red blood cells were lysed using RBC lysis buffer (Invitrogen) and CD4^+^ T cells separated magnetically using mouse CD4^+^ T Cell Isolation Kit (Miltenyi cat. 130-104-454) as per manufacturer’s instructions. Isolated T cells were then labeled with carboxyfluorescein succinimidyl ester (CFSE) Cell Division Tracker kit (BioLegend cat. 423801) as per manufacturer’s instructions. After 8 h co-incubation with OVA protein or bacteria, media was removed from the BMDCs cultures and fresh media containing 200 μg/mL gentamicin (Sigma) was added for 30 min at 37°C. The cells were washed three times with PBS and fresh media was added along with CFSE-labeled CD4^+^ T cells from OT-II mice at a ratio of 1:1 T cells:BMDCs. Plates were incubated at 37°C in 5% CO_2_ for 5 days. T cells were then collected by centrifugation and stained with anti-mouse CD4 Brilliant Violet421 (BioLegend cat. 135510) and anti-mouse CD25 R-phycoerythrin cyanine 7 (BioLegend cat. 101916). T cell proliferation and activation was analyzed using the BD FACSCelesta Analyzer and data was further analyzed using FCS Express version 5 software (*De Novo*).

### Image Stream Analysis

1 × 10^6^ sorted BMDCs were seed in 6-well plates and co-cultured overnight at 37°C with *B. breve* EPS^+^ and *B. breve* EPS^–^ at MOI 10:1. Cultured BMDCs alone were used as negative controls. Cells were washed and incubated with gentamicin (200 μg/mL) for 30 min at 37°C and then washed 3 times in 1 × PBS and stained with CD45 APC, as previously described. Cells were washed twice in wash buffer, fixed with 1% paraformaldehyde and re-suspended in 50 μl of wash buffer. Fixed and stained cell samples were analyzed with an Amnis ImageStreamX MkII cytometer running INSPIRE software version 200.1.620.0 At least 2000 CD45 positive cells were acquired from each experimental sample. Data was analyzed using IDEAS version 6.2.187.0.

### Statistical Analysis

For every experiment at least three biological replicates with three technical replicates per experiment were performed. Statistical analysis was compiled using one way ANOVA with Dunnett’s comparison in GraphPad Prism version 5.03. Statistical comparisons were completed using student’s *t*-tests and *p*-values less than 0.05 were considered significant.

## Results

### Heterogeneous Production of EPS by Human *B. breve* Strains

**FIGURE 1:**
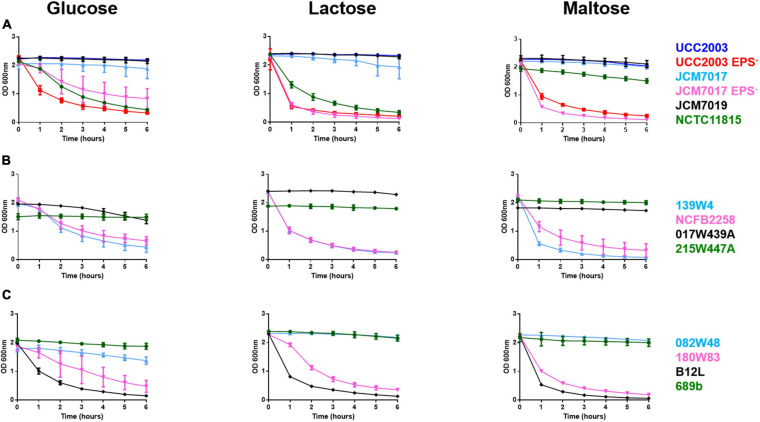
Heterogeneity of EPS production by *B. breve* strains in different carbon sources. *B. breve* strains were cultured overnight, homogenized before OD readings were taken from cultures over 6 h with no disturbance to cultures at all. Bacteria were grown in media containing either glucose, lactose, or maltose as a carbon source. This was done for *B. breve* UCC2003, UCC2003 EPS^–^, JCM7017, JCM7017 EPS^–^, JCM7019 and NCTC 11815 **(A)**, 139W4, NCFB 2258, 215W447A and 017W439A **(B)**, 082W48, 180W83, B12L and 689b **(C)**. Data show the average of three independent experiments.

The EPS status (Table 3) of twelve *B. breve* strains (Table 1) was predicted by *in silico* analyses of *B. breve* genomes for presence of genes required for EPS production. We used the EPS^–^ mutant of *B. breve* UCC2003 as a positive control for an EPS sedimentation assay. Because the carbohydrate source can significantly impact EPS production we screened all *B. breve* strains in media supplemented with different carbohydrates (glucose, lactose, and maltose) (Raftis et al., 2011). *B. breve* UCC2003 and JCM7017 wild type (WT) strains did not sediment but their EPS^–^ mutants did over a period of 6 h (Figure 1A). *B. breve* JCM7019 was a consistent EPS producer while *B. breve* NCTC 11815 sedimented in both glucose and lactose but not in maltose, despite the fact that it was predicted not to produce EPS (Figure 1A and Table 3). *B. breve* strains 017W439A and 215W447A were EPS producers when cultured in all three carbohydrate sources while the 139W4 and NCFB 2258 strains were not (Figure 1B). *B. breve* strains 082W48 and 689b were EPS producers when cultured in all three carbohydrate sources while *B. breve* strains 180W83 and B12L were not (Figure 1C). Comparison between the predicted and actual EPS status of *B. breve* strains (based on EPS sedimentation assay) demonstrated that all predictions were correct except for strain 139W4 which was predicted to produce EPS but actually sedimented *in vitro* irrespective of the carbohydrate source used (Table 3).

**TABLE 3:** EPS status of *B. breve* strains.

**Strain**	**Predicted EPS status**	**EPS status by assay**
UCC2003	Positive	Positive
UCC2003_EPSneg	Negative (mutant)	Negative
JCM7017	Positive	Positive
JCM7017_EPSneg	Negative (mutant)	Negative
JCM7019	Positive	Positive
215W447A	Positive	Positive
017W439A	Unclear	Positive
082W48	Unclear	Positive
689b	Unclear	Positive
NCTC 11815	Negative	Negative
139W4	Positive	Negative
NCFB 2258	Negative	Negative
180W83	Unclear	Negative
B12L	Unclear	Negative

*Strains were predicted in silico to be EPS producers or non-producers based on the presence of pGTF and chain length regulator genes. EPS status in vitro was evaluated using EPS sedimentation assays.*

### Human THP-1 Monocyte Responses to *B. breve* Strains Are MyD88-Dependent

**FIGURE 2:**
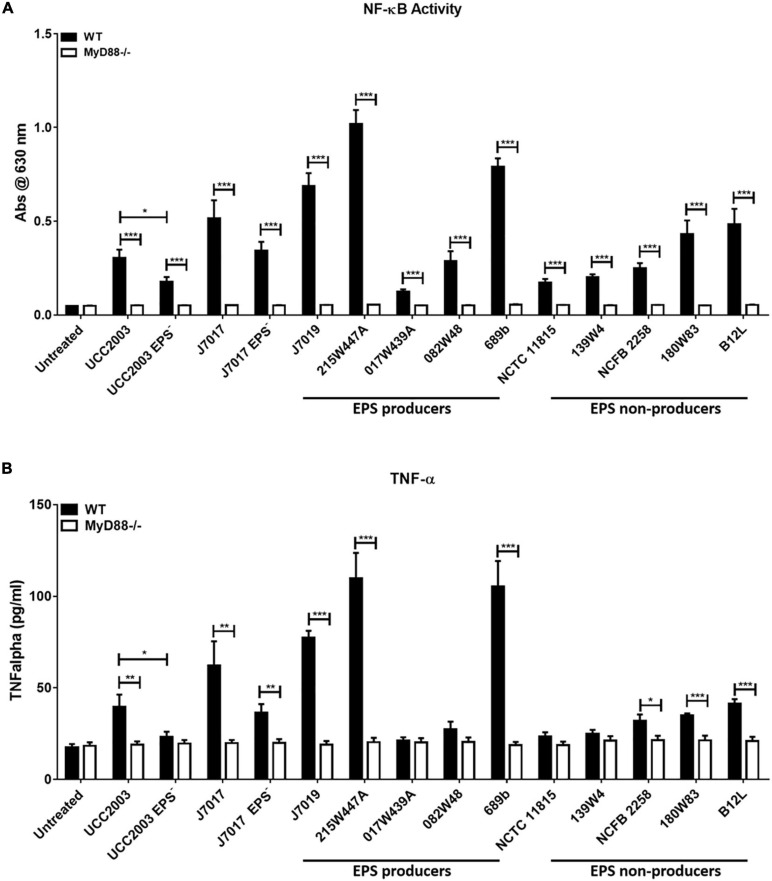
Human THP-1 monocyte responses to *B. breve* strains are MyD88-dependent but EPS-independent. *B. breve* strains were incubated with THP1-XBlue and THP1-XBlue-defMyD reporter cell lines for 24 h. **(A)** NF-κB responses were assessed by measuring reporter gene production, secreted embryonic alkaline phosphatase. **(B)** TNF-α secretion was assessed by ELISA. Data shown is the average of three independent experiments performed in triplicate wells. Data are presented as mean ± SEM. Statistical analysis was assessed using the student *t*-test in GraphPad Prism where *p-value < 0.05, **p-value < 0.005, and ***p-value < 0.0005.

To investigate if EPS production by *B. breve* strains either positively or negatively affected recognition and response to the bacteria by monocytes, the panel of bacteria were co-cultured with WT and MyD88^–/–^ human THP-1 monocyte nuclear factor (NF)-κB reporter cell lines (Figure 2A). NF-κB responses to the panel of *B. breve* strains showed that there was no clear EPS-dependent associated innate immune response. Although strains (*B. breve* 215W447A, 689b, and JCM7019) which stimulated highest activation of NF-κB were all EPS positive, the strain (017W439A) which stimulated lowest activation of NF-κB was also EPS positive (Figure 2A). There was decreased activation of NF-κB by *B. breve* UCC2003 and JCM7017 EPS^–^ isogenic mutant strains compared to their WT parental strains (Figure 2A). Production of TNF-α by THP-1 cells showed a similar trend to NF-κB responses (Figure 2B) for all strains. The lack of response to all strains in the MyD88^–/–^ THP-1 cell line showed that NF-κB activation and TNF-α production was uniformly MyD88-dependent in this cell system (Figure 2). Overall, there was no correlation between EPS status and immune activation of human THP-1 monocytic cells, although all responses to *B. breve* were MyD88-dependent, regardless of strain.

### EPS Modulates Cytokine Response of BMDMs to *B. breve* UCC2003 and JCM7017

**FIGURE 3:**
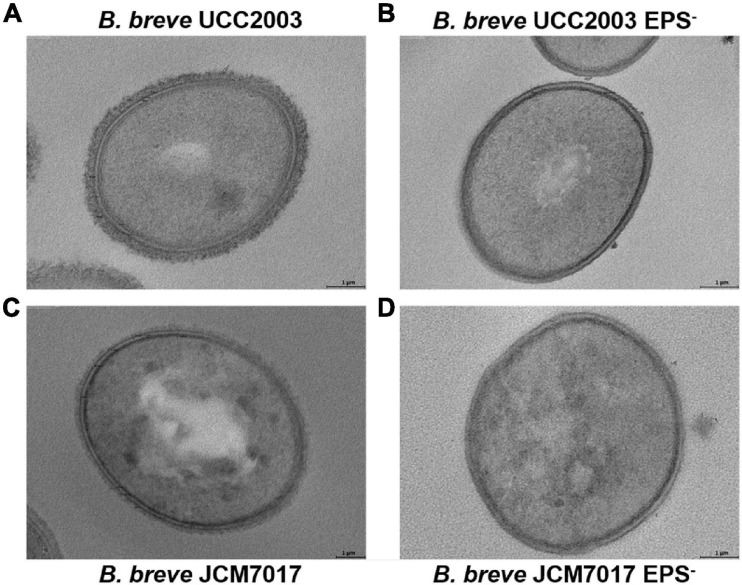
Confirmation of EPS production by *B. breve* UCC2003, JCM7017 and EPS^–^ mutants. Transmission electron microscopy images of *B. breve* UCC2003 **(A)**, *B. breve* UCC2003 EPS^–^ **(B)**, *B. breve* JCM7017 **(C)**, and *B. breve* JCM7017 EPS^–^ **(D)**.

**FIGURE 4:**
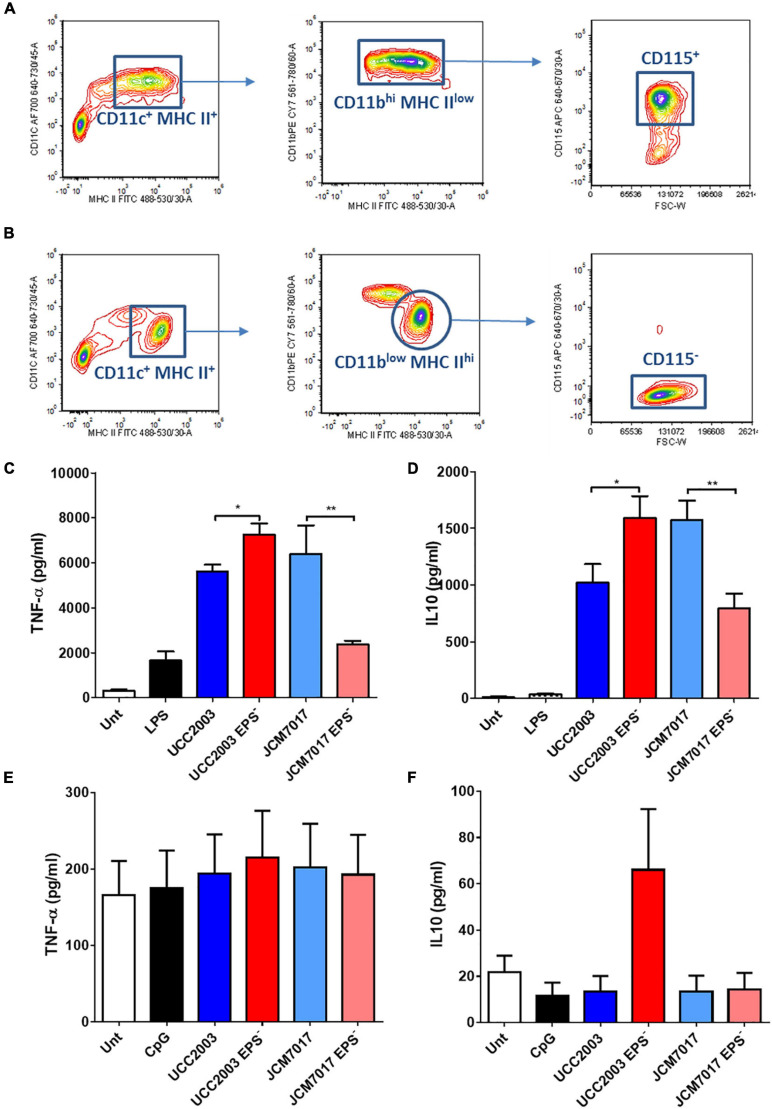
Opposing effects of EPS on murine macrophage cytokine responses to *B. breve* strains. **(A,B)** Gating strategy to identify and sort BMDMs. The bone marrow cells were analyzed and sorted by multi-color flow cytometry. Cells were stained for MHC-II, CD11b, CD11c, and CD115. **(A)** For the adherent cells, MHC-II vs. CD11b contour plots were obtained by gating on MHC-II**^+^**CD11c**^+^** cells. Forward size scattered (FSC) vs. CD115 contour plots were obtained by gating on MHC-II^low^CD11b^high^ cells. CD115**^+^** cells were sorted and considered as pure macrophages population. **(B)** For the suspension cells MHC-II vs. CD11b contour plots were obtained by gating on MHC-II**^+^**CD11c**^+^** cells. FSC vs. CD115 contour plots were obtained by gating on MHC-II^high^CD11b^low^ cells. CD115^–^ cells were sorted and considered as pure dendritic cells population. **(C,D)** TNF-α and IL10 secretion from BMDMs co-cultured with *B. breve* UCC2003, JCM7017 and their isogenic EPS^–^ mutants. **(E,F)** TNF-α and IL10 secretion from BMDCs co-cultured with B. breve UCC2003, JCM7017 and their isogenic EPS^–^ mutants. Cytokines were analyzed by ELISA. Data is the average of three independent experiments performed in triplicates. Statistical analysis was assessed using the student *t*-test in GraphPad Prism where *p-value < 0.05 and **p-value < 0.005.

Because we observed no association between EPS production status and immunomodulation by different *B. breve* strains in THP-1 cells, we investigated the role of EPS on immunomodulation specifically by *B. breve* UCC2003 and JCM7017 using the EPS^–^ isogenic mutants of both strains. EPS production by *B. breve* UCC2003 and JCM7017 parental strains and their respective isogenic EPS^–^ mutants was first confirmed by transmission electron microscopy (TEM) (Figure 3). TEM images showed a noticeably thicker layer of EPS surrounding the WT *B. breve* UCC2003 strain compared with JCM7017 WT strain (Figures 3A,C). In addition TEM confirmed absence of EPS on the surface of both the *B. breve* UCC2003 EPS^–^ and JCM7017 EPS^–^ isogenic mutant strains (Figures 3B,D). To understand how EPS affects macrophage and DC cytokine responses to *B. breve* UCC2003 and JCM7017, primary BMDMs (Figure 4A) and BMDCs (Figure 4B) were co-cultured with the strains and their isogenic EPS^–^ mutants for 24 h. The absence of EPS from *B. breve* UCC2003 enhanced BMDM TNF-α and IL10 cytokine responses, while the absence of EPS from *B. breve* JCM7017 reduced cytokine responses (Figures 4C,D). BMDCs did not produce significant TNF-α or IL10 in response to any bacterial strain (Figures 4E,F).

### EPS Reduces Activation of BMDCs by *B. breve* UCC2003 and JCM7017

**FIGURE 5:**
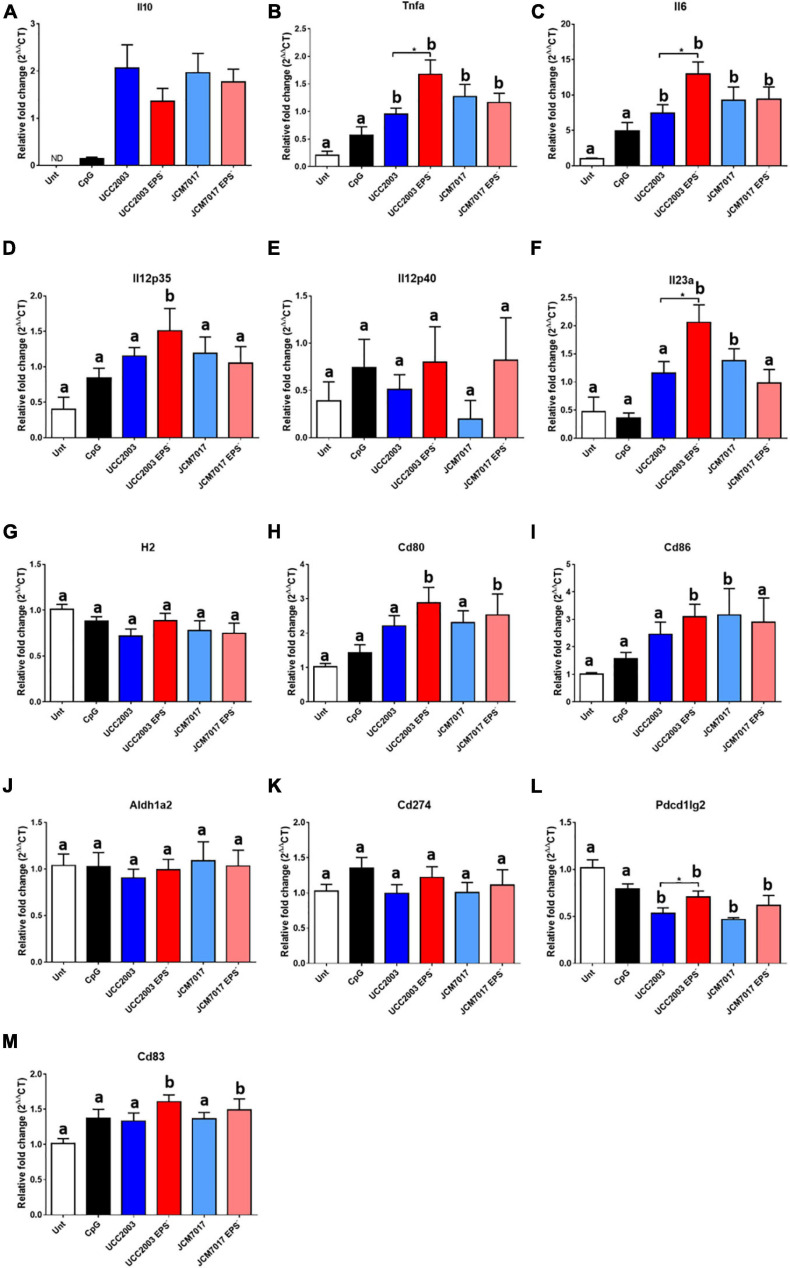
EPS modulates the expression of immunomodulatory genes in BMDCs. Sorted BMDCs were co-cultured or not [untreated (Unt)] with *B. breve* strains as indicated (CpG was used as a positive control) for 4 h. RT-qPCR was carried out and the expression of **(A–F)** cytokine genes **(G–I)** antigen presentation and co-stimulatory genes and **(J–M)** tolerogenic genes were assessed. Expression changes are relative to β-actin. Data show the average of three independent experiments. Gene expression differences between the groups were compared first with the untreated control. Different letters (a and b) indicate statistical difference for each presented gene expression. Letter a indicates no statistical difference between the indicated group and the untreated control, letter b indicates significant differences between the indicated group and the untreated control (*p* < 0.05 of Dunnett’s test). Then, the student *t*-test was used to compare wild type Bifidobacteria (both UCC2003 and JCM7017) with their isogenic EPS mutants (*p-value < 0.05 and **p-value < 0.005).

To further understand the innate immune response of phagocytes to *B. breve* UCC2003 and JCM7017 and how EPS can affect this, we co-cultured sorted BMDCs with the two WT bacterial strains and their EPS^–^ isogenic mutants for 4 h (Figure 5 and Supplementary Table 1) and 8 h (Supplementary Figure 1). We also co-cultured BMDMs with the strains for 8 h (Supplementary Figure 2). RT-qPCR was performed for cytokine genes (*Il10, Tnfa, Il6, Il12a, Il12b*, and *Il23a*), antigen presentation and co-stimulatory genes (*H2/MHC-II, Cd80, Cd83*, and *Cd86*), and immune tolerogenic genes (*Aldh1a2, Cd274*, and *Pdcd1lg2*). *B. breve* UCC2003, JCM7017 strains and their EPS^–^ mutants significantly increased the expression of cytokine genes *Il10, Tnfa, Il6* (Figures 5A–C) relative to untreated BMDCs at 4 h. *B. breve* UCC2003 EPS^–^ also increased expression of both *Il12a/Il12p35* and *Il23a/Il12p19* compared to untreated cells (Figure 5D). However, no significant differences were observed between groups for the expression of *Il12b/Il12p40*, the gene coding for the other subunit of both IL-12p35/p40 and IL-23p19/p40 cytokines which are associated with Th1 and Th17 responses, respectively. No effects were seen on *H2*/MHC-II (Figure 5G) or *Pdcd1lg2*/PDL1 (Figure 5K) expression. Significantly, EPS^–^ mutants of both *B. breve* UCC2003 and JCM7017 – but not EPS WT strains - increased the expression of the T cell co-stimulatory genes *Cd80* (Figure 5H) and *Cd83* (Figure 5M), relative to untreated BMDCs while *Cd86* (Figure 5I) expression was increased by both *B. breve* UCC2003 EPS^–^ and WT JCM7017. The absence of EPS from *B. breve* UCC2003 further enhanced expression of *Tnfa, IL6*, and *Il23a* relative to the WT parental strain (Figures 5B,C,F). No significant differences between the parental WT and EPS^–^ mutant strains of *B. breve* JCM7017 were observed for any of the genes tested. After 8 h co-culture (Supplementary Figure 1 and Supplementary Table 1), all four tested strains continued to significantly enhance *Il10* and *Il6* expression relative to untreated BMDCs (Supplementary Figures 1A,C). Expression of *Tnfa* was also significantly increased by all strains except WT *B. breve* UCC2003 (Supplementary Figure 1B). The absence of EPS from *B. breve* UCC2003 increased *Tnfa* transcription (Supplementary Figure 1B) and decreased Il6 transcription relative to its parental WT strain (Supplementary Figure 1C). Both EPS^–^ mutants induced the expression of *Il23a* compared to untreated (Supplementary Figure 1F) and WT *B. breve* UCC2003 decreased expression of *H2*/MHC-II (Supplementary Figure 1G) and *Cd83* (Supplementary Figure 1M) relative to untreated cells. Importantly, absence of EPS from *B. breve* UCC2003 increased transcription of *Cd83* (Supplementary Figure 1M) at 8 h relative to its parental WT strain. In BMDM after 8 h of co-culture with the strains (Supplementary Figure 2 and Supplementary Table 1), the absence of EPS from *B. breve* JCM7017 increased *Il10* (Supplementary Figure 2A), *Tnfa* (Supplementary Figure 2B), and *Il12a* (Supplementary Figure 2D) transcription. *B. breve* UCC2003 and its associated EPS^–^ mutant showed a similar, non-significant trend for *Tnfa* and *Il12a* gene expression. EPS absence from *B. breve* UCC2003 increased Cd274 expression (Supplementary Figure 2K). No other significant differences were observed in the genes analyzed (Supplementary Figures 2C,E–M). Overall, this gene expression data indicate that the presence of EPS suppresses the expression of cytokine and co-stimulatory genes required for activation and maturation of dendritic cells in response to *B. breve* UCC2003 and JCM7017 strains.

### *B. breve* EPS Blocks Antigen Specific Activation of CD4^+^ T Cells by BMDCs

**FIGURE 6:**
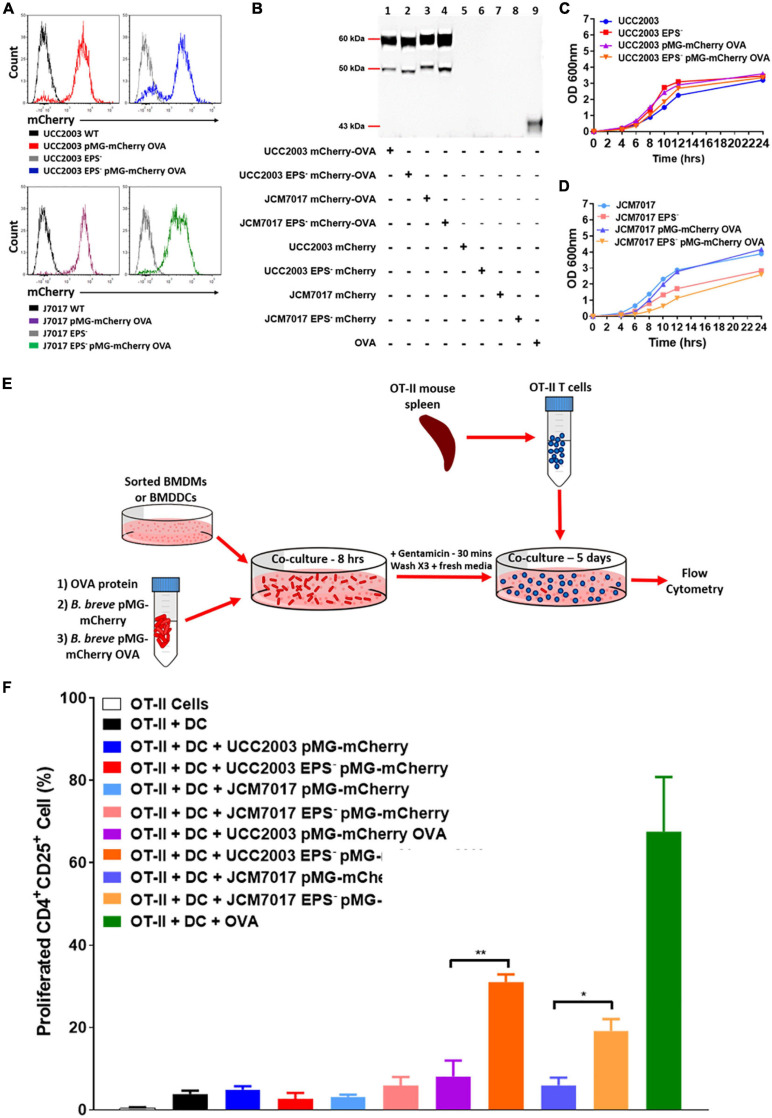
EPS protects from antigen presentation of *B. breve* to OT-II CD4**^+^** T cells by DCs. **(A)** mCherry expression (pMG-mCherry OVA) in the indicated *B. breve* strains by flow cytometry. Histograms were obtained by gating on bacterial cells. Black and gray histograms were obtained from parental *B. breve* UCC2003 and JCM7017 wild type or EPS^–^. Red and blue histograms were obtained from *B. breve* UCC2003 wt and *B. breve* UCC2003 EPS^–^ transformed with the plasmid pMG-mCherry OVA. Purple and green histograms were obtained from *B. breve* JCM7017 wt and *B. breve* JCM7017 EPS^–^ transformed with the plasmid pMG-mCherry OVA. **(B)** Western blot for OVA protein production by pMG-mCherry-OVA and pMG-mCherry transformed strains of *B. breve* as indicated. 0.025 μg of ovalbumin was used as a positive control. **(C,D)** Growth curves of strains transformed or untransformed with the pMG-mCherry-OVA plasmid over 24 h. Growth curves are representative of three experiments. **(E)** Schematic of the antigen presentation assay setup. **(F)** Sorted BMDCs were co-cultured or not with different bacterial strains expressing or not OVA (as indicated) for 8 h. OVA protein was used as positive control. mCherry only expressing bacteria were used as negative controls. Cells were then co-cultured with OT-II CD4**^+^** T cells for 5 days and analyzed by multi-color flow cytometry. T cells were stained for CD4, CD25, and CFSE. Percentage of proliferated CD4**^+^**CD25**^+^** OT-II T cells was quantitated and presented as mean ± SEM. Data is the average of three independent experiments performed in triplicate wells. Statistical analysis was assessed using the student *t*-test in GraphPad Prism where *p-value < 0.05 and **p-value < 0.005.

To further investigate how EPS impacts both the recognition and response of phagocytes to *B. breve* UCC2003 and JCM7017, an antigen presentation experiment was designed (Helft et al., 2015). *B. breve* UCC2003 and JCM7017 and their corresponding isogenic EPS^–^ mutant strains were transformed with pMG-mCherry or pMG-mCherry OVA plasmid. FACS analysis validated expression of mCherry in the transformant bacteria (Figure 6A and Supplementary Figure 3A). Western blotting analysis confirmed that the bacteria expressed mCherry-OVA (Figure 6B and Supplementary Figure 3B). Bacterial growth profiles showed that strains were not seriously affected in any obvious manner by the presence of the plasmid (Figures 6C,D). Antigen presentation assays were set up as illustrated in Figure 6E. Sorted BMDCs were co-cultured with *B. breve* strains transformed or not with pMG-mCherry (used as negative control) or pMG-mCherry-OVA for 8 h. BMDCs were treated with OVA protein as a positive control. After this time, potential extracellular bacteria were killed with gentamicin and BMDCs were washed prior to co-culturing with OVA specific OT-II CD4^+^ T cells for 5 days. T cell activation was determined by examining the numbers of proliferated (CFSE^low^) CD25^+^CD4^+^ T cells. Flow cytometry analysis showed that the absence of EPS from both *B. breve* UCC2003 and JCM7017 significantly increased T cell activation (Figure 6F and Supplementary Figure 4). To investigate if EPS affected uptake of bacteria, BMDCs were co-cultured with *B. breve* UCC2003 strains transformed with pMG-mCherry. After this time, BMDCs were analyzed by Image Stream flow cytometry to identify and quantify uptake of bacteria by cells. *B. breve* UCC2003 EPS^+^ and EPS^–^ were detected inside CD45^+^ (Supplementary Figure 4B). cells and no difference in similarity was observed between cells which had taken up these strains (Supplementary Figure 4C). To investigate persistence of viable *B. breve* UCC2003 and EPS^–^ inside DCs, we co-cultured these bacteria with BMDCs for 2 h. Then, after a gentamicin step to kill extracellular bacteria, we lysed DCs at different time points and plated cell lysates on RCA plates (Supplementary Figure 4D). Colonies of *B. breve* UCC2003 were recovered at significantly higher levels compared to *B. breve* UCC2003 EPS^–^ (Supplementary Figure 4D). Collectively, these results indicate that while EPS does not affect the uptake of bacteria by DCs, it does reduce processing and presentation of bacterial derived OVA antigen and activation of antigen specific CD4^+^ T cells by DCs.

## Discussion

In this paper, we report on the production and immunomodulatory effects of EPS from a collection of human-isolated *B. breve* strains. EPS was produced differentially across a panel of strains and EPS status did not correlate with obvious immunomodulatory effects in a series of immune cell-based assay systems. However, *B. breve* EPS was required for suppression of gene expression associated with and required for full maturation of DCs. Consistent with this, EPS was necessary to prevent processing and presentation of antigen from the strains and antigen-specific activation of CD4^+^ T cells by DCs. Overall, we have identified a new immunomodulatory role for EPS which indicates that EPS is utilized by *B. breve* for immune evasion perhaps as a mechanism to promote host-microbe mutualism.

The panel of *B. breve* strains screened appeared to represent a near equal number of EPS producers and non-producers. Heterogeneity in EPS production has been noted before for bifidobacteria and, taking into account the fact that the genes of EPS biosynthesis loci have a lower GC content than the average bifidobacterial genome, it is proposed that some species and strains acquired the ability to produce EPS by horizontal gene transfer (Hidalgo-Cantabrana et al., 2014; Ferrario et al., 2016). By assessing the phenotypic production of EPS by a panel of *B. breve* strains in different carbohydrate sources, we were able to identify consistent EPS producers and non-producers and compare this phenotypic behavior to *in silico* analysis. The carbohydrate source has previously been shown to impact on EPS production in both lactobacilli (Raftis et al., 2011) and bifidobacteria (Audy et al., 2010). For example lactose induced the highest production of the glycan in *B. longum* subsp. *longum* CRC 002 compared to glucose, galactose, and fructose (Audy et al., 2010) and perhaps this is because lactose itself is a source of glucose and galactose, the two major constituents of EPS in the strain. In addition to carbon source, EPS composition and production can be impacted by other environmental factors such as O_2_, bacterial growth phase, temperature and pH (Degeest et al., 2001). Therefore, it is possible that strains that were phenotypically negative for EPS production in glucose, lactose and maltose-containing medium may be positive under different growth conditions. For example, *B. breve* 139W4 was predicted to produce EPS but did not do so in any of the prepared media under the experimental conditions used. It is possible that it is an EPS producer but that the growth conditions used were not optimal for EPS biosynthesis in this strain. Similarly, this may explain why *B. breve* NCTC 11815 sedimented as an EPS non-producer in glucose and lactose but not in maltose. A screen of bifidobacterial type strains showed that of the 48 (sub)species, 56% were EPS producers, 21% were non-producers and 23% displayed an intermediate phenotype (Ferrario et al., 2016). This variability could also be altered by growth in different media but the mechanistic reasons for this have yet to be explored.

When we assayed for immunomodulatory effects of EPS we found that NF-κB activation in THP-1 monocytes did not correlate with EPS status. This may be due to differences between EPS structures and/or composition of the EPS producers or the presence/absence of other MAMPs involved in triggering NF-κB responses. The fact that there was strain-specific modulation rather than EPS-associated modulation is not entirely surprising as strain-specific effects have been characterized for bifidobacteria (López et al., 2010, 2011, 2012). EPS from *Lactobacillus plantarum* can act in a TLR2-dependent manner to activate signal transducer and activator of transcription 3 (STAT3) independently of NF-κB (Zhou et al., 2018). It is certainly possible that there may be other mononuclear phagocyte responses to these strains which were not investigated here and that are associated with EPS status. In our study the TLR signaling adaptor MyD88 was necessary to activate the NF-κB pathway by all *B. breve* strains tested. This was not entirely surprising considering previous studies linking bifidobacterial immunomodulation to the TLR family and MyD88 (Jeon et al., 2012; Schiavi et al., 2018; Verma et al., 2018; Castro-Bravo et al., 2019). The absence of EPS from both *B. breve* UCC2003 and JCM7017 drove different cytokine responses by BMDMs. While the absence of EPS from *B. breve* UCC2003 increased the production and secretion of IL10 and TNF-α from BMDMs at 24 h the opposite effect was observed for responses to EPS deficient *B. breve* JCM7017 with decreased production and secretion of IL10 and TNF-α detected. It is possible that EPS acts as a cloaking MAMP, reducing the exposure of other bacterial MAMPs on the same cell to PRRs, or even that it shields other MAMPs completely. Any of these scenarios could explain why EPS from *B. breve* UCC2003 appears to modulate more anti-inflammatory effects while EPS from *B. breve* JCM7017 appears to induce more pro-inflammatory effects. It would be useful to understand the composition and structure of *B. breve* UCC2003 EPS in order to compare to the already characterized EPS of *B. breve* JCM7017 (Alhudhud et al., 2018), although TEM images did show significant differences in EPS density between the two strains with *B. breve* UCC2003 having a thicker EPS layer. However, a pure population of sorted BMDCs did not produce IL10 or TNF-α at all to *B. breve* UCC2003 or JCM7017 after 24 h of exposure. BMDCs generated and FACS purified using the approach by Helft et al. (2015) demonstrate a tolerogenic phenotype (Vander Lugt et al., 2017), and represent a pure population of DCs with no contaminating macrophages. This might explain why they did not show an obvious inflammatory response to our *B. breve* strains.

Because we detected EPS-dependent differences in cytokine response of BMDMs to *B. breve* UCC2003 and JCM7017, we analyzed the expression of a panel of immune genes in BMDMs and BMDCs co-cultured with the strains to further understand how EPS can modulate phagocyte immune responses. Our data indicates that both phagocyte populations sensed the presence of the bacteria and responded through PRR-mediated signaling pathways and transcriptional mechanisms to alter gene expression. In BMDCs, the absence of EPS from *B. breve* UCC2003 but not from JCM7017, significantly increased *Tnfa, Il6, and Il23a* expression after 4 h co-culture and decreased, *H2*/MHC-II, *Pdcd1lg2/*PD-L2, and *Cd83* expression after 8 h co-culture. The absence of EPS from *B. breve* JCM7017, but not from *B. breve* UCC2003, significantly increased *Il10* and *Il23a* expression after 8 h co-culture. Significantly, in BMDCs the absence of EPS increased the expression of genes for co-stimulatory molecules (*Cd80, Cd86* for UCC2003 and *Cd83* for both UCC2003 and JCM7017) typically associated with maturation of DCs. This data is consistent with similar suppressive effects of EPS on expression of cytokine genes (*Il12a/Il12p35* and *Il23a/Il12p19*) associated with T cell differentiation and with suppression of T cell activation in our co-culture antigen presentation assay. In BMDMs, the absence of EPS in *B. breve* UCC2003 increased the expression of *Cd274* and the absence of EPS from *B. breve* JCM7017, but not from *B. breve* UCC2003, increased *Il10, Tnfa*, and *Il12a* expression after 8 h. Indeed, we noted an interesting discrepancy in BMDMs between cytokine (*Il10*/IL10 and *Tnfa*/TNF-α) protein data at 24 h and gene expression data at 8 h in response to the EPS deficient JCM7017 strain. While the strain increased *Il10* and *Tnfa* gene expression at 8 h it decreased IL10 and TNF-α cytokine production at 24 h. This curious finding may have an underlying biological mechanism associated with it that is particular to the JCM7017 EPS^–^ strain and the kinetics of BMDM response to this strain. There is evidence in the literature of similar discrepancies and disconnect between cytokine mRNA expression and protein production and secretion. For example in the context of Crohn’s disease it has been reported that despite macrophages showing normal levels and stability of cytokine mRNA intracellular levels of the protein and secretion were abnormally low (Smith et al., 2009). Interestingly, the WT *B. breve* JCM7017 strain did not induce *Il12b/*IL12p40 transcription at all in BMDMs. *Il12b/*IL12p40 encodes a subunit that can be used in conjunction with *Il12a*/IL12p35 to generate the IL-12 cytokine (an important inducer of T_H_1 differentiation) or dimerise with *Il23a*/IL23p19 to create the IL-23 cytokine (an inducer for T_H_17 differentiation). *Il12b/*IL12p40 can alternatively homodimerise to induce macrophage chemotaxis or encourage primed DC migration (Cooper and Khader, 2007). The fact that *B. breve* JCM7017 did not induce expression of the *Il12b/*IL12p40 subunit in BMDMs implies that the bacteria may not support differentiation of T_H_1 or T_H_17 T cells. It also implies that EPS is responsible for controlling this as the EPS^–^ mutant upregulated the expression of *Il12b/*IL12p40 in BMDMs.

We used mCherry-Ova expressing *B. breve* UCC2003 and JCM7017 strains in a co-culture antigen presenting assay to investigate if BMDCs were able to present bacterially expressed antigens to T cells, or if they were just unresponsive to the tested bacterial strains. Although T cell activation was increased by both EPS^–^ mutants expressing OVA, the *B. breve* UCC2003 EPS^–^ mcherry-OVA strain induced stronger T cell activation than the *B. breve* JCM7017 EPS^–^ mcherry-OVA strain. This suggests that EPS is more important for *B. breve* UCC2003 to avoid triggering T cell responses than it is for *B. breve* JCM7017. This observation also ties in with a previous report that the absence of EPS from *B. breve* UCC2003 increased T cell numbers from splenocytes cultured with the bacteria (Fanning et al., 2012). It appears that EPS in different strains may be involved in different functions, possibly because of differences in EPS composition and production. Indeed, higher molecular weight EPS is implicated in better bacterial survival but also reduces traits such as host cell adhesion and aggregation with other bacteria (Yu et al., 2019) and so there may be a trade-off between bacterial fitness and ability to affect cell–cell interactions with host and neighbors. This is possibly the reason for the heterogeneity in EPS production observed, even between EPS producers such as *B. breve* UCC2003 and JCM7017. The absence of EPS from these two strains revealed that it modulates host immune cell responses differently by the two strains but that it also elicits similar functions such as prevention of antigen presentation by BMDCs to T cells. While use of isogenic EPS^–^ mutants offers a potential advantage over the use of purified EPS, observed responses to the bacteria did not clarify if the effect is directly because of the EPS binding a PRR, shielding other MAMP/MAMPs, altering binding affinity of other MAMPs to PRRs, reduction of intra-cellular processing of the bacteria through the phagolysosome or a combination of the above. Certainly EPS is able to shield MAMPs as an EPS^–^ mutant of *Lactobacillus rhamnosus* GG revealed that the bacteria produced sortase-dependent pili on its surface (Lebeer et al., 2009) and a similar observation was made for *B. longum* 105-A (Tahoun et al., 2017). Additionally, it has been shown that while purified EPS from *B. animalis* subsp. *lactis* triggers TLR4 activation, the whole bacteria itself activates TLR2, TLR2/1 and TLR2/6 (Castro-Bravo et al., 2019). Our study shows that while EPS production was heterogeneous amongst human-isolated *B. breve* strains; EPS from *B. breve* UCC2003 and JCM7017 had similar effects on maturation and antigen presentation function of BMDCs. It is important to acknowledge that the biochemical composition and density of EPS observed in both WT strains may contribute to the differences observed in BMDM and BMDCs immune responses. Further detailed biochemical, molecular and cell-based investigations are required to understand the biochemistry and immunological effects of different EPS structures and their effects on dendritic cell biology. Our observations suggest that EPS may contribute to an important immunomodulatory effect of *B. breve* strains by blocking activation and antigen presentation function of DCs. This would allow these strains to manipulate DCs for immunomodulatory effects promoting immune evasion and host-microbe mutualism by these gut symbionts.

## Data Availability Statement

The original contributions generated for this study are included in the article/Supplementary Material, further inquiries can be directed to the corresponding authors.

## Ethics Statement

Use of animals for this study was reviewed and approved by Animal Experimentation Ethics Committee of University College Cork.

## Author Contributions

VR, DV, SM, and KN: conceptualization. AH, VR, PS, SU, AR-V, ME-T, and FB: methodology and writing – original draft. AH, VR, PS, and AR-V: validation. AH, VR, PS, AR-V, JW, and OH: formal analysis. AH, VR, PS, SU, and AR-V: investigation. MV, DV, SM, and KN: resources. AH, VR, PS, and AR-V: data curation. AH, VR, and KN: writing – review and editing. AH, VR, PS, and AR-V: visualization. KN, SM, and DV: supervision. KN: project administration. DV and KN: funding acquisition. All authors contributed to the article and approved the submitted version.

## Conflict of Interest

OH is employed by the company Luminex Corporation which manufactures the Amnis ImageStream flow cytometer used in this study. The remaining authors declare that the research was conducted in the absence of any commercial or financial relationships that could be construed as a potential conflict of interest.
