# Supplementary material for: Bifidobacterium breve Exopolysaccharide Blocks Dendritic Cell Maturation and Activation of CD4+ T Cells
Source: Front Microbiol. 2021 Jun 16;12:653587. doi: 10.3389/fmicb.2021.653587 (PMC8242212; doi:10.3389/fmicb.2021.653587)
Supplement: Supplementary file 1 [file Data_Sheet_1.pdf]

**Supplementary Figure 1.** Sorted BMDCs were co-cultured or not (untreated (Unt)) with *B. breve* strains as indicated (CpG was used as a positive control) for 8 hours. RT-qPCR was carried out and the expression of (a-f) cytokine genes (g-i) antigen presentation genes and (j-m) tolerogenic genes were assessed. Expression changes are relative to β-actin. Data show the average of three wells. Different letters indicate statistical difference in each presented gene expression ($p < 0.05$ of Dunnett's test). The student t-test was used to compare wild type Bifidobacteria with their isogenic EPS mutant (*p value < 0.05 and **p value < 0.005).

**Supplementary Figure 2.** Sorted BMDMs were co-cultured or not (untreated (Unt)) with *B. breve* strains as indicated (LPS was used as a positive control) for 8 hours. RT-qPCR was carried out and the expression of (a-f) cytokine genes (g-i) antigen presentation genes and (j-m) tolerogenic genes were assessed. Expression changes are relative to β-actin. Data is the average of three wells. Different letters indicate statistical difference in each presented gene expression ($p < 0.05$ of Dunnett's test). The student t-test was used to compare wild type Bifidobacteria with their isogenic EPS mutant (*p value < 0.05 and **p value < 0.005).

**Supplementary Figure 3.** a) mCherry expression (pMG-mCherry) in the indicated *B. breve* strains by flow cytometry. Histograms were obtained by gating on bacterial cells. Black and grey histograms were obtained from parental *B. breve* UCC2003 and JCM7017 wild type or $EPS^-$as indicated. Red and blue histograms were obtained from *B. breve* UCC2003 wt and *B. breve* UCC2003 $EPS^-$ transformed with the plasmid pMG-mCherry. Purple and green histograms were obtained from *B. breve* JCM7017 wt and *B. breve* JCM7017 $EPS^-$ transformed with the plasmid pMG-mCherry. b) Loading control using PONCEAU S staining of Western blot for OVA protein production by pMG-mCherry-OVA and pMG-mCherry transformed strains of *B. breve* as indicated.

**Supplementary Figure 4.** a) Sorted BMDCs were co-cultured with variants of *B. breve*, as indicated, expressing or not OVA protein for 8 hr and then co-cultured with CFSE labelled OT-II $CD4^+$ T cells for 5 days and analyzed by flow cytometry. Sorted BMDCs treated with OVA protein alone was used as positive control. T cells were labeled with CFSE to track cell activation. CFSE histograms were obtained after gating on cells with CD4 expression. Number in the quadrants indicates the percentage of the respective activated T cell population in that quadrant. b) Representative images of sorted DCs co-cultured with mCherry expressing *B. breve* UCC2003 and *B. breve* $EPS^-$ (as indicated) and stained for CD45. Numbers in the figures indicate the similarity score values. c) Similarity score for *B. breve* UCC2003 strains (mCherry) as indicated within CD45 cells. d) Enumeration of *B. breve* UCC2003 strains at different time points (as indicated) from BMDCs lysates. The student t-test was used to compare wild type Bifidobacteria with their isogenic EPS mutant (*p value < 0.05 and **p value < 0.005).
