# Supplementary figures and images for: Bifidobacterium breve Exopolysaccharide Blocks Dendritic Cell Maturation and Activation of CD4+ T Cells
Source: Front Microbiol. 2021 Jun 16;12:653587. doi: 10.3389/fmicb.2021.653587 (PMC8242212; doi:10.3389/fmicb.2021.653587)

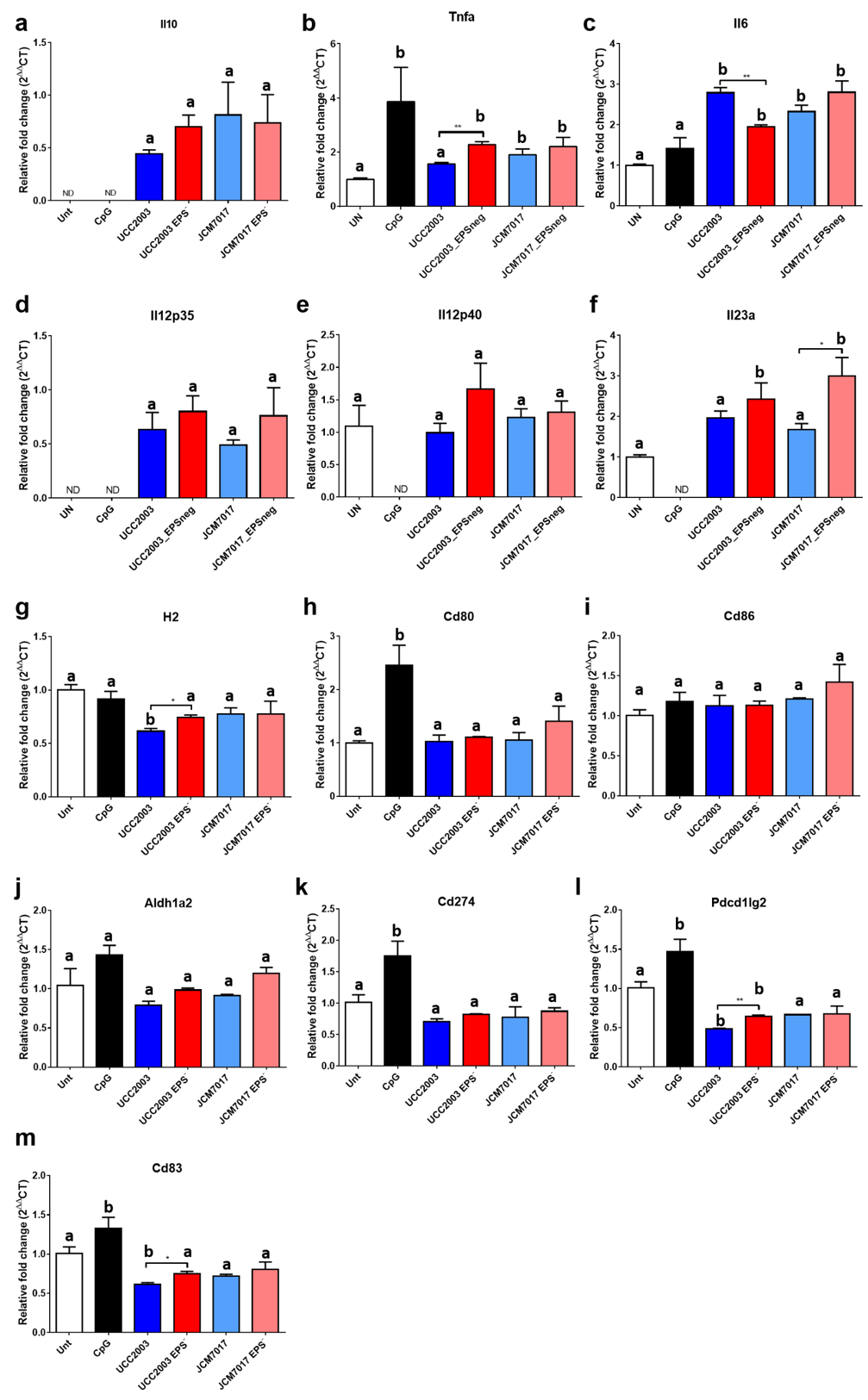

Supplement: Supplementary file 2 [file Image_1.TIF]

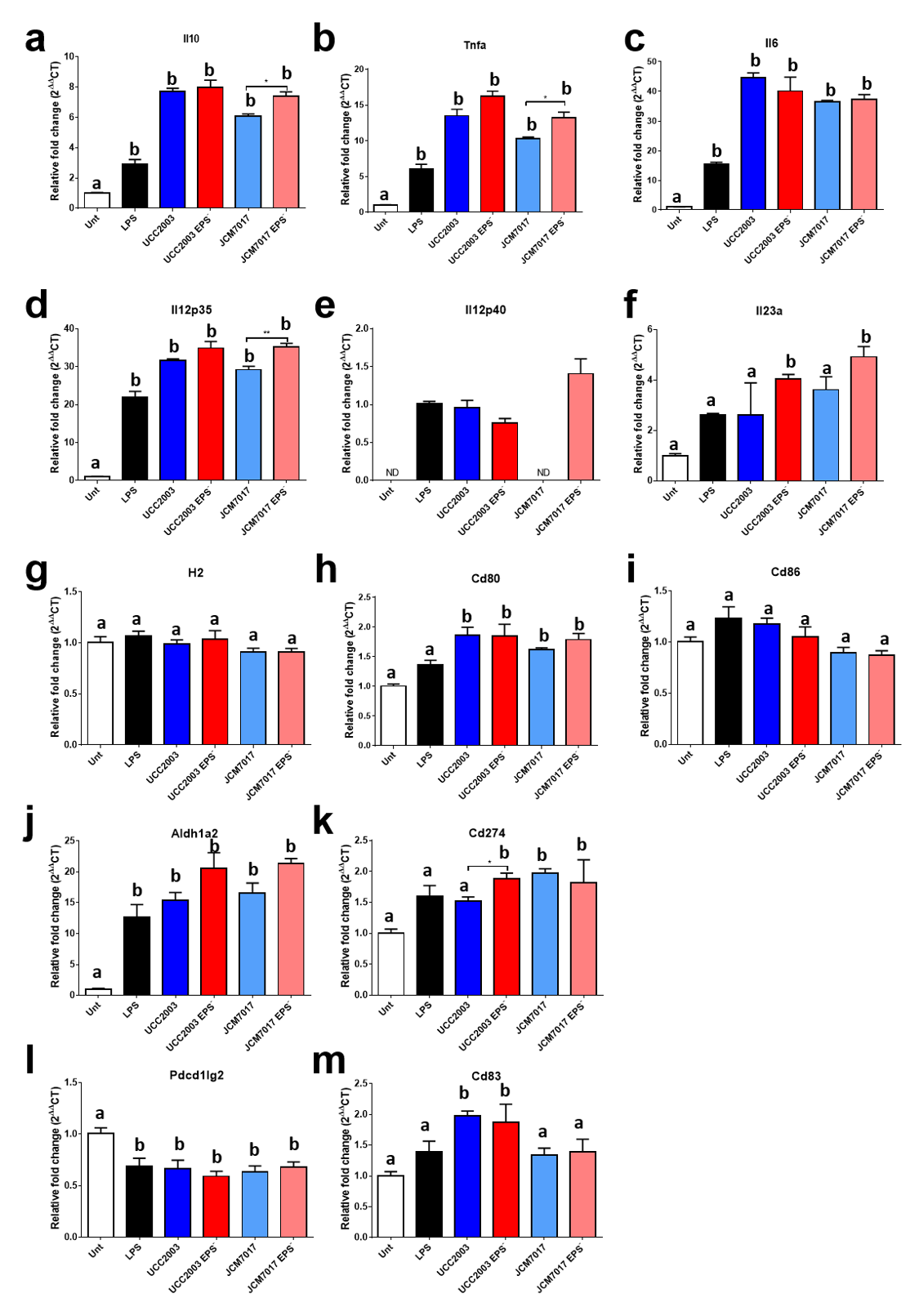

Supplement: Supplementary file 3 [file Image_2.TIF]

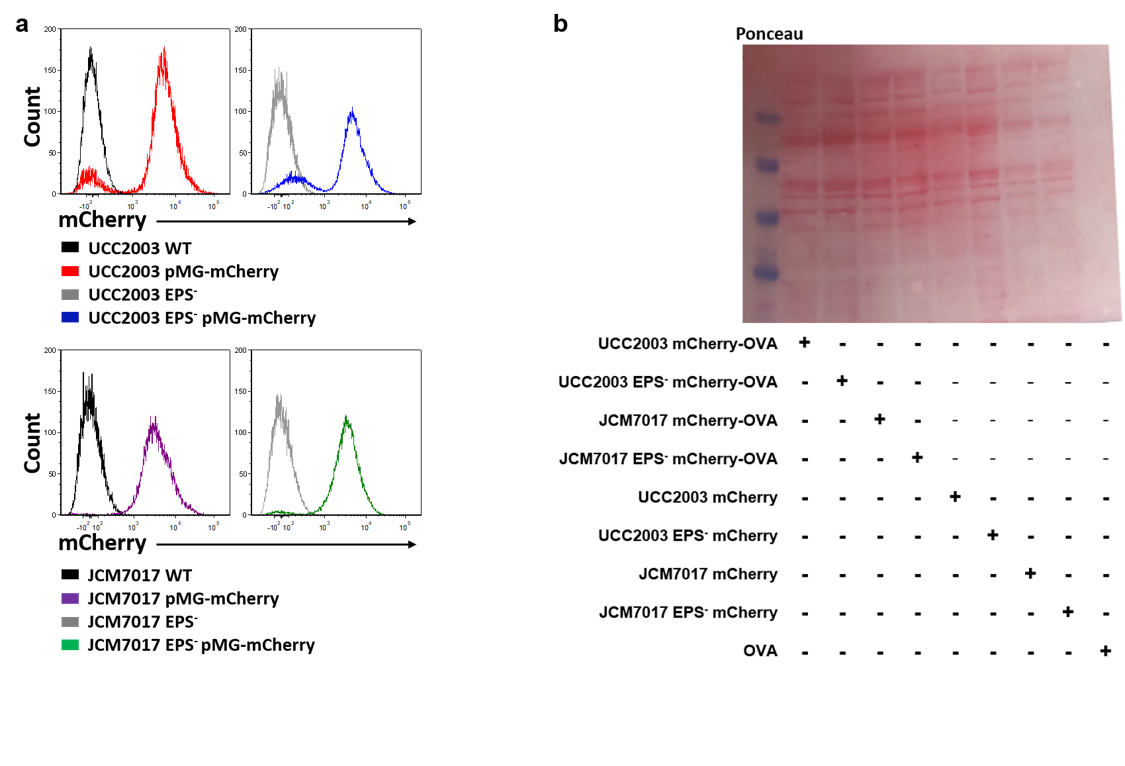

Supplement: Supplementary file 4 [file Image_3.TIF]

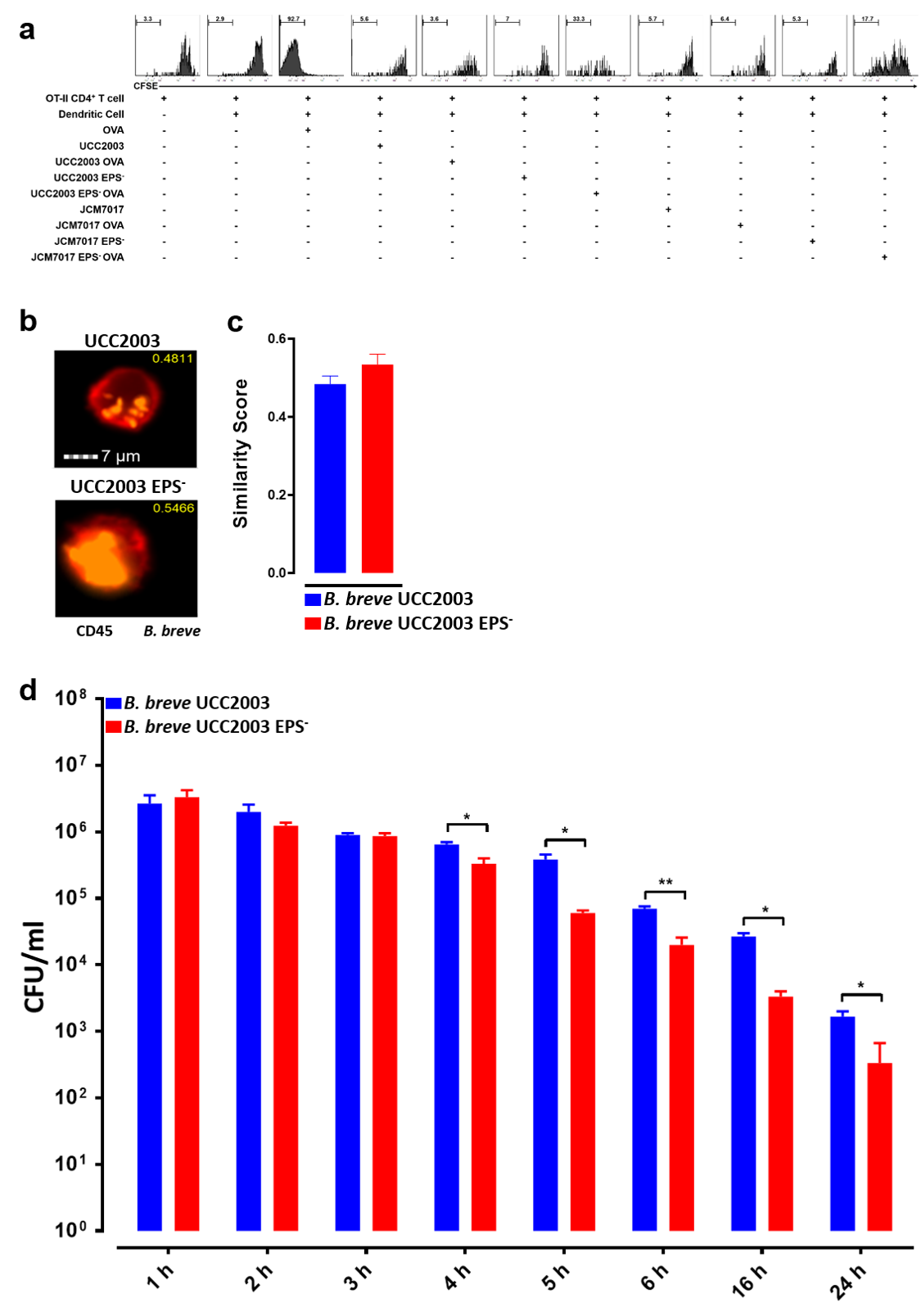

Supplement: Supplementary file 5 [file Image_4.TIF]
